# Psychological evaluation and support in COVID-19 critically ill patients: a feasibility study

**DOI:** 10.1186/s13054-021-03642-1

**Published:** 2021-06-24

**Authors:** Renaud Prével, Julien Coelho, Arthur Orieux, Pierre Philip, Didier Gruson, Stéphanie Bioulac

**Affiliations:** 1 Medical Intensive Care Unit, CHU Bordeaux, 33000 Bordeaux, France; 2 Inserm UMR 1045, Univ Bordeaux, 33000 Bordeaux, France; 3 Service Universitaire Médecine du Sommeil, CHU Bordeaux, 33000 Bordeaux, France; 4 CNRS SANPSY (Sommeil Addiction et Neuropsychiatrie), USR 3413, Univ. Bordeaux, 33000 Bordeaux, France

**To the Editor,**

About 20% of acute respiratory distress syndrome (ARDS) survivors suffer from anxiety, depression or post-traumatic stress disorder (PTSD) [1] with a negative impact on long-term quality of life [2]. Higher rates—38.8%—of psychological sequelae were described in Middle East and Severe Acute Respiratory Syndromes’ outbreaks [3]. Recent data described about 40 to 48% of COVID-19 critically ill patients with post-intensive care disorder or acute stress disorder [4, 5]. The aim of this pilot study is to assess the feasibility of an early psychological evaluation and sustained support in COVID-19 patients admitted to intensive care unit (ICU) and describe their mental health outcomes during a 6-month follow-up. Every COVID-19 survivor was evaluated by a trained clinician psychologist in ICU at invasive ventilation weaning or when conversation was feasible for patients receiving high-flow oxygen. Psychological support was performed as needed according to standard care. Clinician psychologists met the patient and identified if psychological distress symptoms were present (*i.e.* anxious or depressive symptoms, sleep disorder, …). They met the patient as often as required for supportive interventions (to help patients to speak about their emotions and/or fears) and also explained to the patients the care they were given while being sedated. Clinician psychologists systematically met patients or called them by phone as preferred by the patient at day 7, week 6, 12 and 24 after ICU discharge and standardized evaluation occurred at those time points with psychometric evaluation (Hospital Anxiety and Depression Scale (HADS) Posttraumatic Stress Disorder Checklist for DSM-5 (PCL-5) and Insomnia Severity Index (ISI)). Univariate linear mixed regression models were used to identify predictors of mental health in follow-up. Thirty-seven patients were included (81% of men, mean age of 59 (± 11) from March to June, 2020 (Table 1). Three quarters of them (28/37) were intubated for an average length of 40 days (± 7). At discharge from hospital, 20/34 (59%) of them returned home while 14/34 (41%) went to rehabilitation centre. At day 7, 32 patients were evaluated: 5/32 (16%) had significant depression symptoms, 4/32 (13%) significant insomnia symptoms and 3/32 (9%) significant anxiety symptoms and at week 6, they were, respectively, 2/17 (12%), 3/17 (18%) and 2/17 (12%). At week 12, they were, respectively, 5/24 (21%), 3/22 (14%), 3/23 (13%), and 2/25 (8%) to have significant depression, insomnia, anxiety or PTSD symptoms and at week 24, respectively, 2/19 (11%), 2/19 (11%), 4/18 (22%), and 1/18 (6%) (Table 1). Insomnia and anxiety scores did not vary over time (Kruskal–Wallis, respectively, *p* = 0.76 and *p* = 0.95), whereas depression scores decreased at week 24 (Kruskal–Wallis, *p* = 0.04). Cumulative dose of midazolam, cumulative dose of clonidine and length of ICU stay were associated with insomnia in follow-up (respectively regression coefficient *β*: 1.14 [0.44–2.39], *p* = 0.007, *β*: 1.29 [0.24; 2.36], *p* = 0.02 and *β*: 1.02 [0.14; 1.90], *p* = 0.025) (Table 2). Cumulative dose of clonidine was associated with depression in follow-up (*β*: 0.57 [0.11; 1.03], *p* = 0.019). Severity of ARDS could be associated with occurrence of PTSD in follow-up despite non-statistical significance (*p* = 0.059) (Table 2). In conclusion, this study shows that, even in the context of a pandemic situation, it is possible to provide an early and sustained psychological support in critically ill patients. We report lower rates of post-intensive care psychological sequelae than what has previously been reported [4, 5] but the absence of control group prevent from drawing firm conclusions about the impact of psychological intervention. Nevertheless, these results strongly encourage future large randomized controlled studies to assess the efficacy of early psychological evaluation and personalized support in critically ill COVID-19 patients.

**Table 1:** COVID-19 critically ill patients’ socio-demographic, comorbidities and care characteristics

Variables	*n* (%)	Mean ± SD	Median [IQR]
Gender Women Men	37 (100.0%) 7 (18.9%) 30 (81.1%)		
Age	37 (100.0%)	58.8 ± 11.3	59.4 [53.4–63.7]
History of: Sleep disorders Psychiatric consultations On-going depression Past trauma or PTSD Past depression Drug addiction (cannabis) History of: Tobacco use COPD Asthma Chronic hypertension Diabetes Chronic heart condition Chronic coronary disease Chronic kidney disease Pharmacological treatment ACEi Diuretics Proton pump inhibitor Beta-blockers Statins Metformin Fibrates Benzodiazepine Serotonin reuptake inhibitor ARB Aspirin Lithium Anticoagulant Cordarone None	33 (89.9%) 6 (18.2%) 5 (14.7%) 4 (12.2%) 4 (12.2%) 2 (6.1%) 1 (3.0%) 37 (100.0%) 13 (35.1%) 6 (16.2%) 3 (8.1%) 22 (59.5%) 8 (21.6%) 6 (16.2%) 5 (13.5%) 2 (5.4%) 37 (100.0%) 12 (32.4%) 11 (29.7%) 11 (29.7%) 9 (24.3%) 7 (18.1%) 6 (16.2%) 5 (13.5%) 5 (13.5%) 4 (10.8%) 3 (8.1%) 3 (8.1%) 2 (5.4%) 2 (5.4%) 1 (2.7) 7 (18.1%)		
Duration of symptoms before admission to ICU (days)	37 (100.0%)	8.0 ± 2.6	8 [7–9]
Clinical respiratory distress: Yes No	32 (86.5%) 5 (15.6%) 27 (84.4%)		
Thromboembolic event: Yes No	37 (100.0%) 9 (24.3%) 28 (75.7%)		
Acute respiratory distress syndrome Mild Moderate Severe	37 (100.0%) 11 (29.7%) 17 (50.0%) 9 (24.3%)		
Neutrophils/lymphocytes ratio Less than 3.7 More than 3.7	37 (100.0%) 6 (16.2%) 31 (83.8%)		
C-reactive protein (mg/L) Ferritin (µg/L) Lactate Dehydrogenase (UI/L) Albumin (g/L) Fibrinogen (g/L) D-dimers (mg/L)	37 (100.0%) 33 (89.9%) 32 (86.5%) 35 (94.6%) 33 (89.9%) 32 (86.5%)	152 ± 105 2,206 ± 2,043 468 ± 165 20 ± 5 8.5 ± 1.3 5,535 ± 10,478	140 [66–202] 1,470 [941–3,258] 450 [367–537] 21 [16–24] [7.3–9.8] 1,554 [802–5,084]
Oro-tracheal intubation: Yes No	37 (100.0%) 28 (75.7%) 9 (24.3%)		
Duration of intubation (days)	28 (100.0%)	39.9 ± 6.6	39 [38–40]
Duration of sedation (days)	28 (100.0%)	19.0 ± 15.2	14 [9–22]
Cumulative dose of midazolam (mg)	24 (85.7%)	1731 ± 2115	889 [364–2.188]
Cumulative dose of propofol (mg)	22 (78.6%)	23.560 ± 27.083	12.392 [5.060–34.264]
Cumulative dose of morphine (mg)	22 (78.6%)	1459 ± 2491	882 [230– 1.463]
Cumulative dose of clonidine (mg)	22 (78.6%)	2.44 ± 2.18	1.81 [0.85–3.2]
Cumulative dose of levomepromazine (mg)	13 (46.4%)	267 ± 350	173 [0–287]
Use of prone positioning Yes No	28 (100%) 17 (60.7%) 11 (39.3%)		
Use of nitrogen monoxide Yes No	37 (100.0%) 3 (8.1%) 24 (91.9%)		
Use of norepinephrine Yes No	37 (100.0%) 26 (70.3%) 11 (29.7%)		
Maximal dose of norepinephrine (µg/kg/min)	22 (84.6%)	0.30 ± 0.31	0.17 [0.11–0.41]
Cumulative dose of norepinephrine (mg)	23 (88.5%)	85.9 ± 143.4	28.0 [9.5–94.5]
Place of follow-up Home Rehabilitation center	34 (91.9%) 20 (58.8%) 14 (41.2%)		
Psychometric evaluation at day 7 Significant depression Significant insomnia Significant anxiety	32 (86.5%) 5 (15.6%) 4 (12.5%) 3 (9.4%)		
Psychometric evaluation at week 6 Significant depression Significant insomnia Significant anxiety	17 (45.9%) 2 (11.8%) 3 (17.7%) 2 (11.8%)		
Psychometric evaluation at week 12 Significant depression Significant insomnia Significant anxiety Significant PTSD symptoms	24 (64.9%) 5 (20.8%) 22 (59.5%) 3 (13.6%) 23 (62.2%) 3 (13.0%) 25 (67.6%) 2 (8.0%)		
Psychometric evaluation at week 24 Significant depression Significant insomnia Significant anxiety Significant PTSD symptoms	19 (51.4%) 2 (10.5%) 2 (10.5%) 18 (48.6%) 4 (22.2%) 1 (5.6%)		

*ACEi* angiotensin-converting enzyme inhibitor, *ARB* angiotensin II receptor blocker, *COPD* chronic obstructive pulmonary disease, *ICU* intensive care unit *PTSD* post-traumatic stress disorder

**Table 2:** Univariate mixed model with random intercept of factors associated with the occurrence of psychological sequelae during follow-up

	*β*	[95%] CI	*p* value
*Predicting insomnia in follow-up*			
History of sleep disorders	4.24	[− 0.54;9.08]	0.082
Acute respiratory distress syndrome			0.099
Mild	Reference	Reference	
Moderate	1.32	[− 2.46;5.09]	
Severe	4.87	[0.35;9.34]	
Cumulative dose of morphine (for 1000 units)	0.74	[− 0.06;1.53]	0.069
Cumulative dose of midazolam (for 100 units)	**1.41**	**[0.44;2.39]**	**0.007**
Cumulative dose of clonidine (for 1 unit)	**1.29**	**[0.24;2.36]**	**0.020**
Maximal dose of norepinephrine	− 2.78	[− 11.5;5.98]	0.520
Length of ICU stay (for 10 days)	**1.02**	**[0.14;1.90]**	**0.025**
*Predicting anxiety in follow-up*			
History of sleep disorders	0.98	[− 1.87;3.78]	0.488
Acute respiratory distress syndrome			0.206
Mild	Reference	Reference	
Moderate	0.75	[− 1.39;2.94]	
Severe	2.36	[− 0.30;4.97]	
Cumulative dose of morphine (for 1000 units)	0.15	[− 0.45;0.76]	0.612
Cumulative dose of midazolam (for 100 units)	0.02	[− 0.12;0.07]	0.537
Cumulative dose of clonidine (for 1 unit)	0.43	[− 0.12;0.98]	0.118
Maximal dose of norepinephrine	− 2.61	[− 7.64;2.44]	0.301
Length of ICU stay (for 10 days)	0.46	[− 0.07;0.99]	0.091
*Predicting depression in follow-up*			
History of sleep disorders	1.19	[− 2.09;4.41]	0.464
Acute respiratory distress syndrome			0.823
Mild	Reference	Reference	
Moderate	0.75	[− 1.89;3.42]	
Severe	0.81	[− 2.43;3.99]	
Cumulative dose of morphine (for 1000 units)	0.05	[− 0.60;0.69]	0.883
Cumulative dose of midazolam (for 100 units)	0.02	[− 0.05;0.09]	0.588
Cumulative dose of clonidine (for 1 unit)	**0.57**	**[0.11;1.03]**	**0.019**
Maximal dose of norepinephrine	− 2.82	[− 8.11;2.54]	0.292
Length of ICU stay (for 10 days)	0.39	[− 0.23;1.01]	0.208
*Predicting post-traumatic stress disorder in follow-up*			
History of sleep disorders	8.36	[− 1.60;18.4]	0.100
Acute respiratory distress syndrome			0.059
Mild	Reference	Reference	
Moderate	0.55	[− 7.13;8.17]	
Severe	9.81	[1.06;18.5]	
Cumulative dose of morphine (for 1000 units)	0.21	[− 1.77;2.17]	0.826
Cumulative dose of midazolam (for 100 units)	0.16	[− 0.04;0.35]	0.103
Cumulative dose of clonidine (for 1 unit)	0.93	[− 1.14;3.01]	0.363
Maximal dose of norepinephrine	5.71	[− 9.65;21.0]	0.449
Length of ICU stay (for 10 days)	1.54	[− 0.37;3.42]	0.109

Bold values indicate statistically significant association

*β*: regression coefficient of the linear mixed model with random intercept

*CI* confidence interval, *ICU* intensive care unit

## Data Availability

The datasets analysed during the current study are available from the corresponding author on reasonable request.

## Abbreviations

ARDS: Acute respiratory distress syndrome
HADS: Hospital Anxiety and Depression Scale
ICU: Intensive care unit
ISI: Insomnia Severity Index
PCL-5: Posttraumatic Stress Disorder Checklist for DSM-5
PTSD: Post-traumatic stress disorder
